# Deep Learning Approaches for Detecting COVID-19 From Chest X-Ray Images: A Survey

**DOI:** 10.1109/ACCESS.2021.3054484

**Published:** 2021-01-25

**Authors:** Hanan S. Alghamdi, Ghada Amoudi, Salma Elhag, Kawther Saeedi, Jomanah Nasser

**Affiliations:** 1 Information Systems DepartmentFaculty of Computing and Information TechnologyKing Abdulaziz University 37848 Jeddah 21589 Saudi Arabia; 2 Faculty of MedicineKing Abdulaziz University 37848 Jeddah 80215 Saudi Arabia

**Keywords:** Chest x-ray, coronavirus, COVID-19, deep learning, radiological imaging

## Abstract

Chest X-ray (CXR) imaging is a standard and crucial examination method used for suspected cases of coronavirus disease (COVID-19). In profoundly affected or limited resource areas, CXR imaging is preferable owing to its availability, low cost, and rapid results. However, given the rapidly spreading nature of COVID-19, such tests could limit the efficiency of pandemic control and prevention. In response to this issue, artificial intelligence methods such as deep learning are promising options for automatic diagnosis because they have achieved state-of-the-art performance in the analysis of visual information and a wide range of medical images. This paper reviews and critically assesses the preprint and published reports between March and May 2020 for the diagnosis of COVID-19 via CXR images using convolutional neural networks and other deep learning architectures. Despite the encouraging results, there is an urgent need for public, comprehensive, and diverse datasets. Further investigations in terms of explainable and justifiable decisions are also required for more robust, transparent, and accurate predictions.

## I. Introduction

Early diagnosis of the coronavirus disease (COVID-19) is essential to reduce the spread of the virus and provide care for preventing complications. The daily increments in COVID-19 cases worldwide and the limitations of the current diagnostic tools impose challenges in identifying and managing the pandemic. Researchers worldwide are actively participating to find effective diagnostic procedures and accelerate the development of a vaccine and treatments. As of the writing of this paper, three diagnostic procedures are commonly used: blood tests, viral tests, and medical imaging [1]. Blood tests detect the presence of severe acute respiratory syndrome coronavirus 2 (SARS-CoV-2) antibodies in the blood. However, the reliability of this test in diagnosing COVID-19 is as low as 2% or 3% [2]. Viral tests detect the antigens of SARS-CoV-2 using samples from the respiratory tract. The rapid diagnostic test (RDT) is a type of antibody detection test that is fast and can produce results in 30 min. However, the availability of RDT test kits is limited, and its effectiveness depends on the sample quality and the time of onset of illness. Furthermore, the test can yield false positive results because it does not distinguish COVID-19 from other viral infections; therefore, it is not recommended for diagnosing COVID-19 [3]. Another commonly used viral test is reverse transcription polymerase chain reaction (RT-PCR). RT-PCR is the gold-standard tool used as the first-line screening choice [4]. However, large-scale studies have found that the test result sensitivity ranges between 50–62% [4]. This implies that an initial negative RT-PCR result can be obtained. Therefore, to ensure the correctness of the test result for diagnosis, multiple RT-PCR tests are performed over a 14-day observation period. In other words, an RT-PCR negative result for a suspected case of COVID-19 is only considered as a true negative when there are no positive RT-PCR results after multiple tests have been taken over the 14-day observation period [5]. This can be frustrating for the patient and costly for the healthcare authorities owing to the shortage of RT-PCR test kits in several countries [6].

**FIGURE 1.**
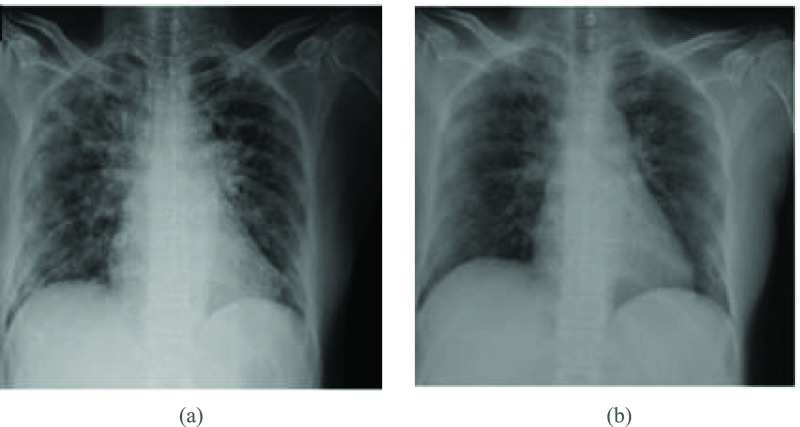
CXR findings: (a) GGO and (b) GGO with consolidation [8].

Because COVID-19 targets the respiratory system, chest radiology scans are an important tool for diagnosis and early management. Chest X-rays (CXR) have been used as a first-line diagnostic tool in Italy and various other countries [7]. The condition of the lungs can be effectively detected using radiology scans along with the different stages of illness or recovery [8]. Radiologists have recorded a range of abnormalities found in the radiology scans of COVID-19 patients. Fig. 1 shows two examples of COVID-19 features in CXR images, namely, bilateral GGO and bilateral and multifocal GGO with consolidation.

CXR is a widely available tool in most clinical settings; it is less time-consuming in terms of patient preparation and immediate diagnosis. Consequently, CXR can be used for patient triage, deciding the priority of patient treatments, and utilizing medical resources.

In the medical imaging domain, deep learning (DL) techniques have been used to improve the performance of image analysis significantly [9], [10]. For example, DL has been successfully applied to microscopy images [11], brain tumor classification [12], MRI images [13], and retinal photographs [14].

Convolutional neural networks (CNNs) are commonly used for medical imaging [15], [16]; they have various architectures and applications. Therefore, since the first few months of the pandemic, DL approaches have been extensively explored for diagnosing COVID-19 from radiology photographs. In this paper, we review the latest research contributions of the application of DL for the detection of COVID-19 from CXR images by comparing the existing DL technologies, highlighting the challenges, and identifying the required future investigations.

**FIGURE 2.**
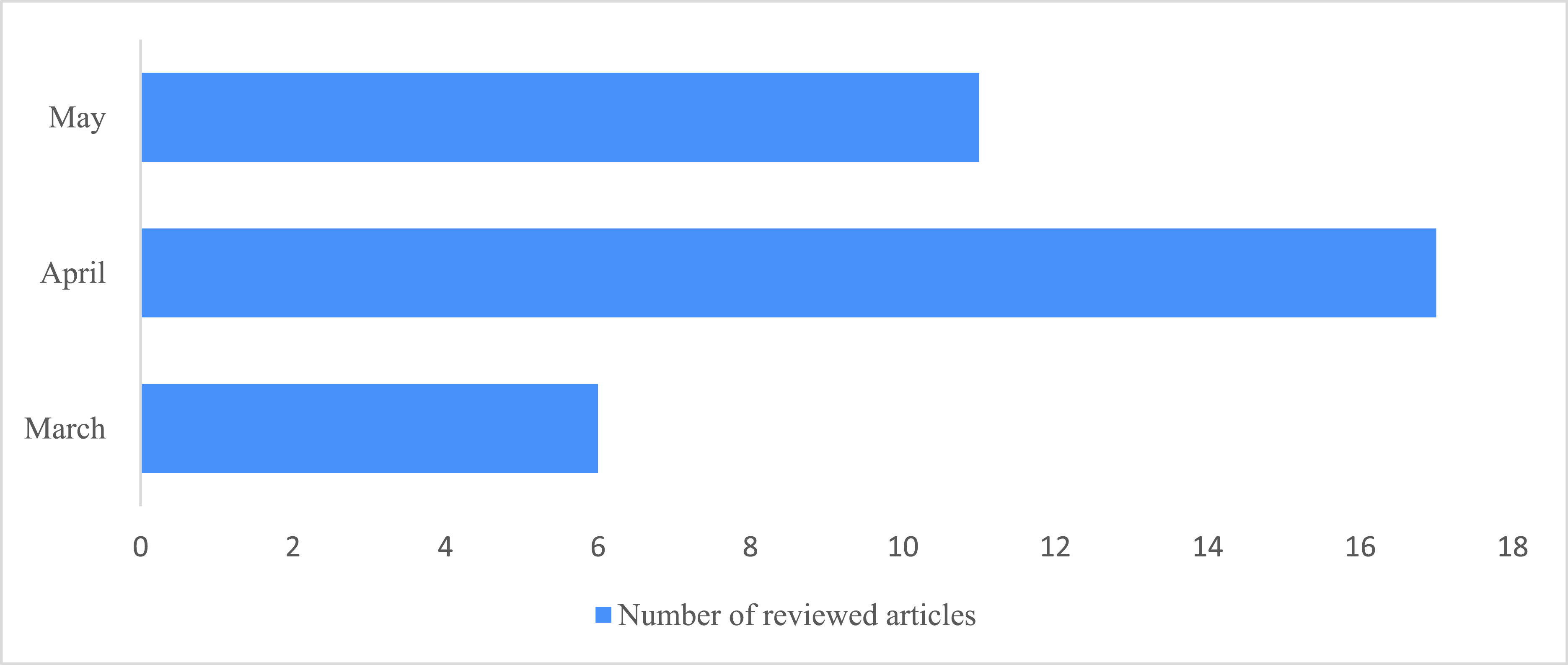
Distribution of the reviewed articles in the months of 2020.

To understand how CNNs and other DL architectures could facilitate the diagnosis of COVID-19 via CXR images, this paper reviews and critically assesses the preprint and published reports made available between March and May 2020 on this topic. The articles were found in several common research databases, such as PubMed, ScienceDirect, Springer, IEEE, ACM, Scopus, ArXiv, and MedRxiv. The keywords used in the search included “transfer learning,” “convolutional,” “deep learning,” “radiograph,” “chest x-ray,” “CXR,” “COVID,” and “Coronavirus;” this list was regularly updated since the beginning of this study. We reviewed the paper abstracts and excluded those studies that considered DL for computed tomography images and those that used traditional machine learning algorithms. When articles from multiple resources overlapped, only the most recent articles were considered. Fig. 2 shows a histogram of the distribution dates of the papers included in this review.

Section II provides a detailed review of DL applications for COVID-19 CXR image analysis, including the architectures used and CXR datasets employed. Section III presents the methodology analysis and performance comparisons of various application of deep learning modeling. Section IV highlights the current challenges and discusses the findings of this survey, including public dataset establishment, model optimization, model uncertainty, and the unexplained black-box decisions made by the DL models. Finally, the paper is concluded with some suggestions for further investigations in Section V.

## II. Literature Survey

We reviewed 34 articles investigating the use of DL models to examine CXR images with SARS-CoV-2 viral infections. The vast majority of the studies (71%) implemented transfer learning using publicly available CNN architectures trained on the ImageNet dataset. These architectures with their parameters and hyperparameter settings are publicly available [17]. However, 29% of the studies went beyond using off-the-shelf tools and implemented novel architectures. In the following subsections, we provide a general overview of the main approaches and datasets that were used in the research works reviewed in this survey.

### A. Classification Task Formulation

**FIGURE 3.**
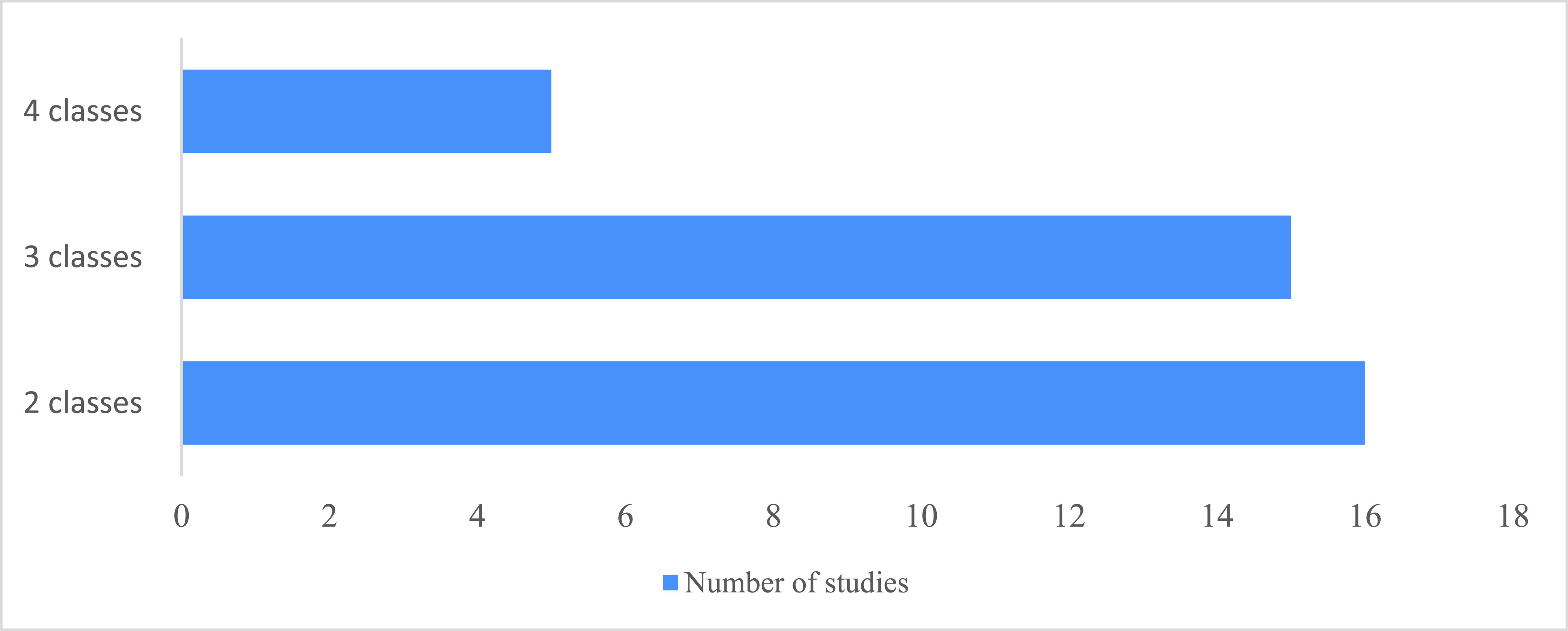
Distribution of studies according to classification task formulation.

The COVID-19 detection results are given by classifying the CXR images into 2–4 classes, i.e., binary or multi-class classification. Each class represents one or more labels: “healthy,” “no finding,” “bacterial pneumonia,” “viral pneumonia,” or “COVID-19.” Two-class classification is called binary classification, and its results include the COVID-19 label and either of the following labels: “healthy,” “no finding,” “bacterial pneumonia,” or “viral pneumonia.” The three-class results include “COVID-19,” “healthy or no finding,” and “pneumonia.” The four classes results include “COVID-19,” “healthy or no finding,” “bacterial pneumonia,” and “viral pneumonia.” Most of the reviewed research used two or three classes. Fig. 3 shows the number of reviewed studies grouped by the number of classes used in the classification task.

### B. Datasets

In the reviewed articles, 14 different datasets were used. A summary of these datasets is presented in Table 1. Each row specifies the reference, dataset name, a brief description, and whether the dataset contains COVID-19 samples. According to our survey, COVID-19 Image Data Collection [18] is the mostly cited dataset. It contains images extracted from various online publications and websites in an attempt to provide AI researchers with COVID-19 photographs to develop deep learning-based models. Each image in this dataset is accompanied by a set of attributes such as sex, age, date, survival, and clinical notes.

**TABLE 1:** Description of the COVID-19 Datasets Used in the Reviewed Research

No.	Reference	Name(s)	Description	COVID-19 Samples
1	[18]	COVID-19 Image Data Collection	Public CXR images of COVID-19 collected from various online sources with ongoing updates.	$\surd $
2	[20]	Figure 1 COVID-19 CXR Dataset Initiative (COVIDx)	48 CXR images of COVID-19	$\surd $
3	[61]	ActualMed COVID-19 CXR Dataset Initiative	N/A	$\surd $
4	[62]	COVID-19 Radiography Database	2,905 CXR images with 219 COVID-19 positive cases	$\surd $
5	[63]	Japanese Society of Radiological Technology	105 CXR images of COVID-19 cases	$\surd $
6	[22] sometimes referenced as [64]	ChestX-ray8 ChestX-ray14	Contains 112,120 frontal-view CXR images of 30,805 patients labeled with 14 thoracic diseases. This dataset is also called the NIH CXR dataset and RSNA Pneumonia Detection Challenge Dataset.	$\times $
7	[65]	SIRM COVID-19 Database	68 COVID-19 CXR images	$\surd $
8	[19]	Radiopaedia.org	An open-source website that shares radiographs. As of the publication of this work, it contained 20 COVID-19 CXR images.	$\surd $
9	[66]	ChexPert Dataset	224,316 chest CXR images of 65,240 patients labeled for 14 thoracic diseases.	$\times $
10	[67]	Twitter COVID-19 CXR Dataset/ COVID-19 Chest Imaging at thread reader	135 CXR images showing SARS-CoV-2 viral infection. This dataset is provided by a cardiothoracic radiologist from Spain through his Twitter account.	$\surd $
11	[68] taken from [69]	Pediatric Pneumonia Chest X-ray	5,856 pediatric CXR pneumonia images of children aged 1–5 years, labeled as “pneumonia” or “normal.”	$\times $
12	[69] or [70] sometimes referenced as [23]	Labeled Optical Coherence Tomography (OCT) and Chest X-Ray Images for Classification/Kaggle Chest X-Ray Images (Pneumonia)	A dataset composed of OCT and 5,863 CXR images of normal, viral pneumonia, and bacterial pneumonia cases.	$\times $
13	[71]	Open-I Repository	Open access biomedical image search engine provided by the US National Library of Medicine—CXR images of COVID-19 can be found with the relevant publication.	$\surd $

Table 2 reveals the quantitative values related to the datasets including number of records, image resolution, types, and the URL. The URL is the address to access the dataset, which offers opportunity for researchers to reuse the dataset, compare the results, and enhance their knowledge. Table 2 shows that Github.com and Kaggle.com are the mostly used platforms to store and make datasets available online.

**TABLE 2:** COVID-19 Dataset Sizes, Formats, and Download Links

No.	Reference	# Images	Resolution	Format	URL
1	[18]	315	$4248\times3480$	JPG	https://github.com/ieee8023/covid-chestxray-dataset
2	[20]	48	various	JPG, PNG	https://github.com/agchung/Figure1-COVID-chestxray-dataset
3	[61]	N/A	various	PNG	https://github.com/agchung/Actualmed-COVID-chestxray-dataset
4	[62]	2,905			https://github.com/tawsifur/COVID-19-Chest-X-ray-Detection
5	[63]	105	$4020\times4892$	N/A	http://db.jsrt.or.jp/eng.php
6	[22] sometimes referenced as [64]	112,120	$1024\times1024$	DICOM PNG	https://www.kaggle.com/c/rsna-pneumonia-detection-challenge/data
7	[65]	68	various	JPEG	https://www.sirm.org/category/senza-categoria/COVID-19/
8	[19]	N/A	various	JPEG and others	Radiopaedia.org
9	[66]	224,316	$320\times320$	N/A	https://stanfordmlgroup.github.io/competitions/chexpert/
10	[67]	135	2012$\times $2012	JPG	https://twitter.com/ChestImaging
11	[68] taken from [69]	5,856	various	JPEG	https://www.kaggle.com/andrewmvd/pediatric-pneumonia-chest-xray
12	[69] or [70] sometimes referenced as [23]	5,863	various	JPEG	https://www.kaggle.com/paultimothymooney/chest-xray-pneumonia
13	[71]	N/A	various	various	https://openi.nlm.nih.gov/

It is worth noting that, some studies referred to the same dataset by a different name; for instance, the COVID-19 Image Data Collection [18] has been referred to as the “Montreal database collection” in some studies, although the original name is used in most of the reviewed research. This dataset is composed of images from different sources, including Radiopaedia.org [19], the Italian Society of Medical and Interventional Radiology (SIRM) COVID-19 Database [65], and http://Figure1.com [20]. Note that these resources were used in some studies with the COVID-19 Image Data Collection [18], creating the possibility of data duplication in the combined datasets.

The COVID-19 Radiography Database [21], which is the winner of the COVID-19 Dataset Award, is a dataset composed from six different sources, including the SIRM COVID-19 Database, COVID-19 Image Data Collection, Twitter COVID-19 CXR Dataset, RSNA Pneumonia Detection Challenge dataset [22], Kaggle CXR Images (Pneumonia) [23] and other CXR images from multiple published studies. Rahman *et al.* [21] stated that they only gathered images from published work and addressed redundancy by comparing the CXR images from different studies with those in the COVID-19 Image Data Collection to eliminate duplications. Other datasets, such as the datasets obtained from Peshmerga Hospital, Erbil, Kurdistan [24], were obtained from local hospitals and are not publicly available.

### C. Transfer Learning

Transfer learning has been widely adopted in medical imaging applications [15], [25]. Transfer learning is beneficial in situations where the training examples are insufficient for training a model from scratch. Tajbakhsh *et al.* [25] demonstrated that a pre-trained CNN with adequate fine tuning might outperform or perform as well as a CNN trained from scratch. Consequently, and because of the limited training datasets, transfer learning has been actively explored for the detection of COVID-19 from CXR images.

In this survey, the reviewed works that utilized transfer learning can be categorized into three groups. In the first group, a pre-trained CNN on a large-scale natural image dataset was used to initialize the weights of a new network that will be trained on the target CXR data. For instance, models trained on ImageNet were used in [26], [27], and [28].

The second group consists of studies wherein some of the early layers of the pre-trained model on large-scale natural image dataset were frozen and their weights kept unchanged while the final layers were finetuned [29]. This practice is based on the fact that the early layer features are more generic (e.g., edges), whereas the later-layer features are more specific to a particular task or dataset [17]. Examples of works that implemented the finetuning approach for the radiological photographs can found in [26], [27], [30], and [31].

In the third group of studies, transfer learning was implemented using a model pre-trained on a similar target domain; for example, Afshar *et al.* [31] trained a model on a radiography dataset of patients with and without pneumonia. They then trained the model further on COVID-19 CXR images. The studies in this group claimed that the use of models trained on ImageNet is not the best option for medical applications because the source (natural images) and target domains (e.g., CXR images) are different [30], [31]. However, the results of a comparative study by Cheplygina [29] did not fully support this assumption; the study examined 12 articles that compared the use of medical images to natural images in transfer learning in medical imaging research. The goal of the study was to determine which source images are better in medical transfer learning tasks: natural images such as ImageNet or medical images. Among the 12 articles examined, the study found that six articles supported each claim, i.e., each claim is supported equally; therefore, the study concluded that the selection of the model and source data depends on the task at hand among other factors.

### D. CNN Architectures

In recent years, CNN architectures [9] have managed to achieve human expert-level performance in a wide range of complex visual tasks, including medical image assessment and pathology detection. Numerous CNN architectures have been proposed in the literature since the very first successful CNN in 1998. Known as LeNet and developed by Yann LeCun, it was widely used for handwritten digit recognition [32]. Compared to current models, LeNet is considered to be a shallow architecture; it contains three convolutional, two average pooling, and two fully connected layers. In the following subsections, we briefly describe the CNN architectures used in the reviewed studies along with their usage and results for COVID-19 detection from CXR images.

#### 1) AlexNet

AlexNet [33] is similar to LeNet, but it is deeper and contains three stacked convolutional layers. AlexNet won the 2012 ILSVRC challenge and achieved a top-five error rate of 17%. To overcome the overfitting problem, the authors used a dropout regularization technique and data augmentation in AlexNet.

Razzak *et al.* [34] used AlexNet for the binary and multiclassification of COVID-19 cases. They achieved a test set accuracy of 97.04% for COVID-19/healthy binary classification and 63.27% for COVID-19/healthy/bacterial pneumonia/viral pneumonia multiclassification tasks.

Kumar and Kumari [35] used AlexNet as a feature extractor to feed a support vector machine (SVM) classifier and achieved an accuracy of 93.0%. Abbas *et al.* [36] also used AlexNet for the feature extraction of three classes, namely, normal, COVID-19, and SARs. However, in their work, they proposed that a class decomposition layer should be added to partition each class into multiple sub-classes. These subsets were reassembled to produce the final predictions. They used AlexNet to find features for the proposed decomposition layer and achieved an accuracy of 95.12% for their proposed model DeTraC.

#### 2) GoogleNet

GoogleNet [37] won the 2014 ILSVRC challenge and achieved a top-five error rate of 6.67%. This network is significantly deeper than the previous CNNs; in addition to the pooling and convolutional layers, GoogleNet contains an inception module (IM). This module acts as a small network and can learn cross-channel correlations (depth-wise) along with spatial correlations. It consists of six convolutional layers: four $\mathrm {1\times 1}$ convolutional layers, one max pooling layer, and one concatenation layer [37]. The IM serves as a bottleneck layer and induces several advantages. First, it enables the training of significantly deeper models while reducing the number of learnable parameters by nearly ten times. Second, the output of an IM is configured to be smaller than its input in terms of the number of feature maps. Thus, the IM reduces the dimensionality. Third, an IM can capture complex patterns at multiple scales along with the spatial and depth dimensions. Other variants of GoogleNet have been proposed using slightly different inception components, and they have achieved better performances. Examples include Inception-V2, Inception-V3 [38], Inception-ResNet, and Inception-v4 [39].

Razzak *et al.* [34] used GoogleNet in a similar manner as AlexNet, i.e., for the binary and multiclassification of COVID-19 cases, and the test set accuracy improved to 98.15% for COVID-19/healthy binary classification and 75.51% for COVID-19/healthy/bacterial-pneumonia/ viral-pneumonia multiclassification tasks.

Similarly, Kumar and Kumari [35] used GoogleNet as AlexNet for feature extraction and achieved an accuracy of 93% using an SVM classifier.

#### 3) VGGNet

VGGNet [40] was proposed by the Visual Geometry Group (VGG) at Oxford University and was the runner up of the 2014 ILSVRC challenge; it achieved a top-five error rate of 7.3%. With a total of 16 or 19 convolutional layers, VGGNet has the advantage of architectural simplicity [40]. However, it used three times more parameters than AlexNet [38].

Moutounet-Cartan () [41] evaluated five different CNN architectures followed by a flat multi-layer perceptron. They found that VGG16 yielded the best test accuracy of 70.6% for detecting COVID-19 cases over three classes of COVID-19/ no findings/other pneumonia, followed by VGG19 with an accuracy of 70.2%; meanwhile, the InceptionResNetV2 and InceptionV3 architectures yielded significantly lower accuracies of 45.7% and 47.7%, respectively. Rahaman [42] also found that VGG19 achieved the highest testing accuracy of 89.3% compared to the 14 other deep CNN architectures.

Kumar and Kumari [35] also used VGG16 and VGG19 to extract the features of COVID-19 to feed the SVM with the final accuracies of 92.7% and 92.9%, respectively.

#### 4) ResNet

ResNet [43] introduced a residual learning component to the CNN architecture. The residual unit (RU) consists of a regular layer with a skip connection. The skip connection allows the input signal of a layer to traverse the network by connecting it to the output of that layer. Thus, the RUs enabled the training of an extremely deep model of 152 layers, which won the 2015 ILSVRC challenge and achieved a top-five error rate of under 3.6%. Other variants of ResNet have 34, 50, and 101 layers.

**FIGURE 4.**
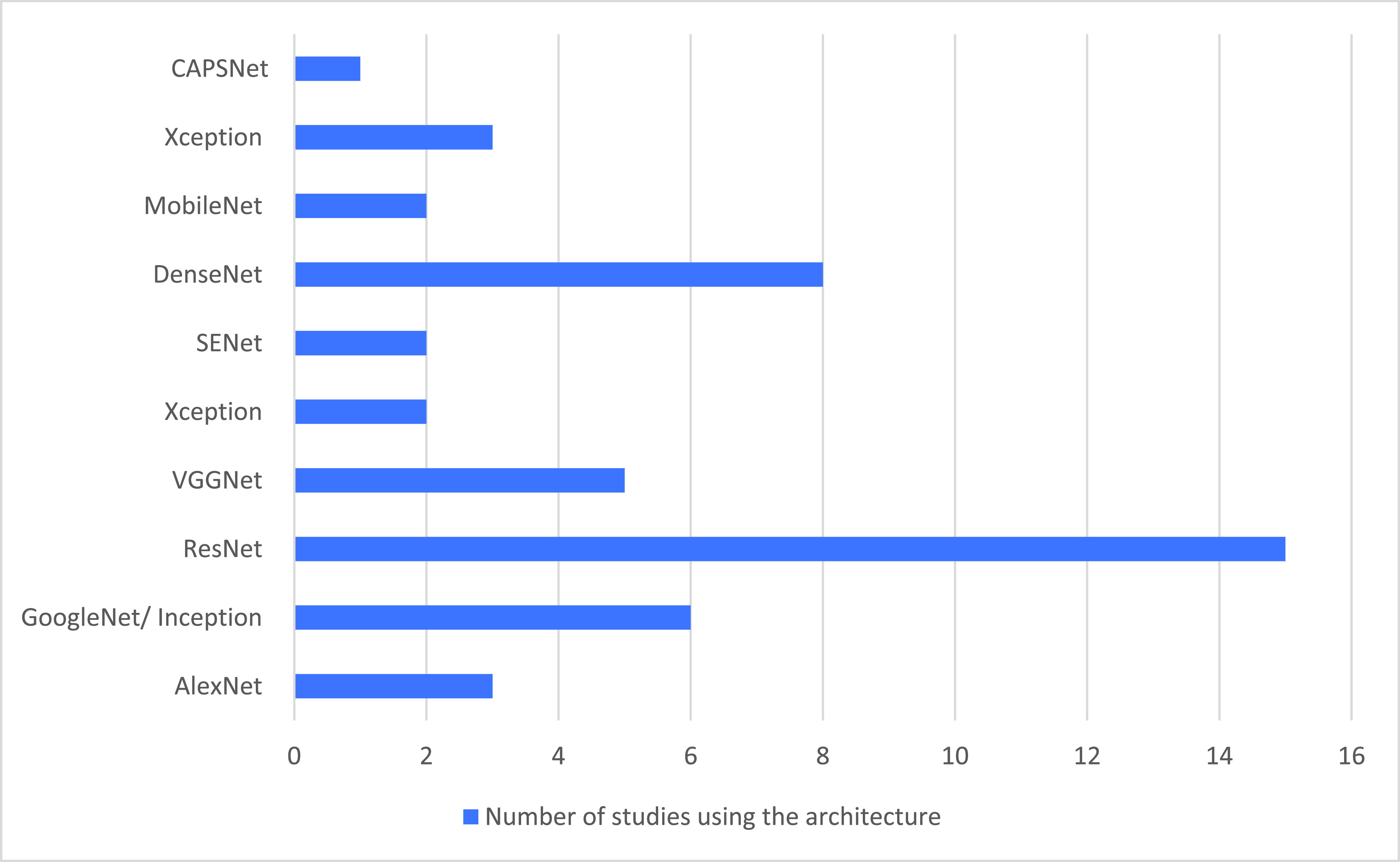
CNN architectures used in the reviewed work.

As shown in Fig. 4, ResNet was the most widely utilized CNN architecture in the reviewed papers. Minaee *et al.* [28] applied ResNet18 and ResNet50 on an imbalanced dataset of 100 COVID-19 images and 3000 non-COVID images; they achieved a sensitivity of 98% for both architectures. ResNet50 yielded an accuracy of 89.2% for detecting COVID-19 from CXR in [44]; Kumar and Kumari [35] used three variants of ResNet with an SVM classifier including ResNet18, which provided an accuracy of 91%. ResNet50 provided an accuracy of 95% and ResNet101 provided an accuracy of 89.2%.

#### 5) Xception

Xception [45] was proposed by Francois Chollet, and it outperformed Inception-V3 on a huge image classification task comprising 350 million images and 17,000 classes. In contrast to the IM of GoogleNet, the main concept of Xception involves learning the cross-channel and spatial patterns separately. Thus, in Xception, the IM is replaced with a special layer called depth-wise separable convolution. Compared with a traditional convolutional layer, the separable convolution layer has fewer parameters, lower computational cost, and an overall better accuracy of 79% in comparison to Inception-V3 (78.2% accuracy) on the ImageNet dataset [45].

El-Din Hemdan [46] used Xception to diagnose COVID-19 in normal patients. Xception obtained the highest precision among the deep learning classifiers for detecting only positive COVID-19 cases; however, it exhibited significantly worse performance in classifying the normal cases. In [35], Xception yielded a precision and sensitivity of 0.830 and 0.894 in identifying COVID-19 patients, respectively.

#### 6) SENet

The Squeeze and Excitation Network (SENet), proposed by Hu *et al.* [47], was the winner of the 2017 ILSVRC Challenge with a top-five error rate of 2.251%. SENet extends the GoogleNet IM and ResNet architectures and boosts their performances. SENet introduced a new component, namely, the SE block. This block has been added to every IM or RU in ResNet. The extended versions are called SE-Inception and SE-ResNet, respectively. The SE block consists of three simple layers, a layer for global average pooling across the feature maps, a squeeze dense layer, and a dense layer. The output of the SE block recalibrates the feature maps generated by the IM or RU in a way that downscales the irrelevant feature maps and retains the relevant features. Note that the SE block focuses on the cross-channel patterns, instead of the spatial patterns, and learns the features that are active together. In other terms, it learns the objects in the images that usually appear together.

Razzak *et al.* [34] used SENet for COVID-19 detection and achieved accuracies of 98.89% and 94.39% for binary and multiclassification tasks, respectively.

Minaee *et al.* [28] used SENet as a ResNet variant on a highly imbalanced dataset and achieved similar sensitivity of 98%, but improved specificity of 92.9% in comparison to 90.7% and 89.6% of the ResNet models, respectively.

#### 7) DenseNet

DenseNet [48] connects each layer to every other forward layer in the network. Therefore, instead of $L$ connections between $L$ layers in the regular CNN architecture, DenseNet contains $L(L\mathrm {+1)/2}$ layer connections. All subsequent layers use the feature maps generated by any layer in the network, which enables all layers to reuse and propagate features, including the final ones. On ImageNet, DenseNet achieved a top-five error rate of 6.12%; however, it requires fewer parameters and less computational cost than other state-of-the-art CNN architectures, such as ResNet.

As shown in Fig. 4, DenseNet with its variants is the second most used architecture in the reviewed studies. Razzak *et al.* [34] used DenseNet and achieved accuracies of 98.75% and 93.46% for binary and multiclassification, respectively. In [28], the application of DenseNet resulted in similar sensitivity as the aforementioned architectures, but a lower specificity of 75.1%. Kumar and Kumari [35] and El-Din Hemdan *et al.* [46] used DenseNet201 for COVID-19 detection and achieved accuracies of 93.8% and 90%, respectively.

#### 8) MobileNet

MobileNet [49] is a lightweight CNN designed for mobile and embedded vision systems. MobileNet utilizes depth-wise separable convolution to generate a lighter architecture and introduces two trade-off hyperparameters to balance the latency and accuracy. MobileNet has been shown to perform well across a wide range of applications [49].

MobileNet was used for COVID-19 detection from the CXR images in [34] and [46], achieving accuracies of 96.30% and 60%, respectively.

#### 9) ShuffleNet

Other advanced CNN architectures include ShuffleNet [50], which outperformed MobileNet on the ImageNet classification task. Compared with AlexNet, ShuffleNet achieved a speedup of 13 times with comparable accuracy. To reduce the computational cost, ShuffleNet introduces channel shuffle and pointwise group convolution operations.

From the reviewed studies, Alqudah *et al.* [51] used ShuffleNet for the automatic extraction of features, which were then fed to four different classifiers: Random Forest, Softmax, SVM, and KNN. The accuracies achieved by these classifiers with the ShuffleNet features were 80%, 99.35%, 95.81, and 99.35%, respectively.

### E. Other DL Architectures

In this survey, we found two additional DL architectures that are not based on the basic CNN components but were also suggested for COVID-19 detection, namely, autoencoder and Capsule Network (CapsNet).

An autoencoder [52] is a type of unsupervised neural network. The features learned by autoencoders, also called codings, are a compressed representation of the input image. This makes the autoencoders useful for feature extraction, dimensionality reduction, and pre-training supervised models.

Autoencoders were used by Khobahi *et al.* [53] for the detection of COVID-19 and achieved an accuracy of 93.50%.

CapsNet was first introduced by Hinton *et al.* [54]. It contains a special arrangement of neurons, called a capsule; a traditional neuron outputs a scalar and a capsule output a vector. The length of the vector represents the existence of an object in the image and the vector orientation represents the object properties [55]. CapsNet has been demonstrated to be a powerful and promising tool for managing the variations in the orientation, position, and lighting.

CapsNet was used by [31] for identifying the COVID-19 cases. By pre-training with a dataset of X-ray images, CapsNet in [31] provided an accuracy of 98.3%.

## III. Methodology Analysis

As discussed in previous sections, various DL architectures (CNNs in particular) have been proposed for the detection of COVID-19 from CXR images in a relatively short time. In this section, we provide in-depth insights about the reviewed studies.

### A. Datasets

Table 3 details some well-known CNN architectures applied for COVID-19 classification along with the datasets used. All reviewed studies used publicly available datasets, except the studies of Iqbal Khan *et al.* [90] and Gomes *et al.* [24].

**TABLE 3:** Well-Known CNN Architectures Used for the Detection of COVID-19

Research study	Datasets	CNN architecture(s) employed
[72]	[18], [65], [22]	InceptionV3
Deep-COVID [28]	[18], [66]	ResNet18, ResNet50, SqueezeNet, DenseNet121
[73]	[18], [23]	Bayesian ResNet50V2
[74]	[18], [22], [66], [68], [66], [64]	VGG 16, InceptionV3, Xception, DenseNet121, NASNet-mobile
[31]	[18], [23]	CapsNet
[26]	[18], [22]	ResNet18
DeTraC [36]	[18], [63]	AlexNet, ResNet18
[75]	[18], [64]	ResNet50V2, Xception
[51]	[18], [64], [65], [19], [23]	AOCT-Net, MobileNet, ShuffleNet
[34]	[18] [23]	AlexNet, SqueezeNet, GoogLeNet, VGG, MobileNet, ResNet18, ResNet50, ResNet101, DenseNet
[53]	[18], [64]	Autoencoders, ResNet18
[76]	[18], [65], [23]	DenseNet161
[57]	[23]	ResNet152
[41]	[18], [20]	VGG16, VGG19, InceptionResNetV2, InceptionV3, Xception
[6]	[18], [69]	RestNet50
[77]	Locally collected from Hospital San Gerardo, Monza, Italy, and IRCCS Policlinico San Donato, Italy	RestNet10
[46]	[18]	VGG19, DenseNet121, InceptionV3, ResNetV2, Inception, ResNet-V2, Xception, MobileNetV2
[35]	[18], (Winner of the COVID-19 Dataset Award) [21]	AlexNet, DenseNet201, GoogleNet, InceptionV3, ResNet18, ResNet50, ResNet101, VGG16, VGG19, XceptionNet, InceptionResNetV2
[44]	[18], [65], [69]	ResNet50, VGG16
[27]	[18], [23]	ResNet50, InceptionV3, InceptionResNetV2
[79]	[20]	SqueezeNet
[66]	DenseNet121
[80]	[20]	DenseNet121
[42]	[18] [23]	VGG16, VGG19, ResNet50, ResNet50V, ResNet101, ResNet101V2, ResNet152, ResNet152V2, DenseNet121, DenseNet169, DenseNet201, MobileNetV1, XeptionNet, InceptionV3, InceptionResNetV2
[82]	[18]	CNN

Most of the studies combined datasets to enlarge the training set. However, due to the limited number of available COVID-19 samples, an imbalance problem occurs, which is one of the major challenges. The table also illustrates that most of the studies employed a number of different architectures to compare the classification result or to build an ensemble model to achieve better performance.

The dataset collected by Cohen *et al.* [18] was used in more than 85% of the articles surveyed here. The collection of CXR images in the dataset of Cohen *et al.* was obtained from online publications rather than from original medical sources, which may have reduced the image quality and led to undesirable learning models. This dataset also does not provide lesion or infected area annotation. Visual annotation would help to get more insights regarding the reasons of the prediction decisions made by human experts and would help in comparisons with deep learning architectures.

The disagreement between human annotators should be also provided to allow better model evaluation.

### B. Deep Learning Models Constructing

The review indicates that transfer learning was preferred by most researchers, and broad interest in this approach continues. DL, particularly transfer learning, enables rapid model development while outperforming other approaches. ResNet [43], DenseNet [48], Inception [38], and VGG [40] are among the most utilized pre-trained architectures. A properly trained transfer learning model will usually outperform a model trained from scratch. The smaller the dataset, the better the performance. However, as the number of labeled COVID-19 CXR images is currently limited, common pre-trained models such VGG or ResNet, with millions of parameters, can easily overfit the training data. Thus, particular attention should be paid towards choosing the appropriate metrics for evaluation and for selecting appropriate and representative testing data.

Some studies investigated more than one version of the same base CNN, VGG16, and VGG19. As shown in Fig. 4, most authors employed a ResNet followed by DenseNet. In the iplementation of transfer learning, a new model requires a pre-trained network chosen from among the widely adopted networks that are trained on the ImageNet dataset as a starting point. Although most studies exploited architectures trained on ImageNet, Duchesne *et al.* [58] and Bassi and Attux [59] applied transfer learning using ChexNet [60]. ChexNet is a 121-layer dense CNN model (DenseNet) trained on the ChestX-ray14 dataset [22], which contains 112,120 frontal-view CXR images labeled with 14 different thoracic diseases, including pneumonia. In ChexNet, the final DenseNet fully connected layer is replaced with a fully connected layer whose output is produced using a nonlinear sigmoid function. The weights of the network were initialized with weights from a model pre-trained on ImageNet.

Table 4 summarizes the contributions and novelty of some articles reviewed in this survey along with the datasets they used for evaluation. The approaches applied varied from building frameworks and models from scratch to exploiting transfer learning along with some advanced feature extraction methods. For example, in [53], autoencoders were used for feature extraction. In [26], the authors combined anomaly detection scores with classification scores in the last layer of the network. The anomaly detection component was used to generate a large anomaly score of CXR images with COVID-19. The authors demonstrated that this hybrid approach outperforms other individual task learning models. The authors of [61] proposed to automatically generate a new deep architecture named COVID-Net, which has been tailored particularly for COVID-19 CXR image classification. COVID-Net is open source along with its COVIDx dataset, comprising 13,975 CXR images. The main advantage of this approach is that the architectural design choices made by generative synthesis can achieve a balance between multiple objectives such as performance and computational cost. This approach can be further investigated and applied to other medical image classification tasks by specifying the requirements such as the desired sensitivity and specificity.

**TABLE 4:** Articles Proposing Novel Methods for COVID-19 Detection via CXR Images

Research study	Dataset(s)	Contribution(s)
[30]	[18], [65], [19]	The authors built a model from scratch trained on more than 100,000 CXR images and used transfer learning to overcome the problem of limited number of COVID-19 samples.
[26]	[18], [22]	Anomaly detection was used in the last layer to classify COVID-19 CXR images. This layer generates a scalar anomaly score that assigns statistically significantly large classification scores and anomaly scores to CXR images with COVID-19.
COVID-Net [61]	[18], [22], (Winner of the COVID-19 Dataset Award) [21]	The authors used GenSynth to generate a deep CNN tailored for COVID-19 by specifying the design requirements, thereby obtaining a sensitivity of 80% and PPV of 80%.
DeTraC [36]	[18], [63]	Original classes were decomposed into several sub-classes using K-nearest neighbors; transfer learning (ImageNet pre-trained ResNet) was applied, and the final classification was refined using the error-correction criteria.
CheXNet [59]	[18], [65], [23], (Winner of the COVID-19 Dataset Award) [21]	ChexNet was trained with transfer learning twice.
COVID-DA [83]	[18], [22], [21]	A domain adaptation method was proposed to transfer the domain knowledge from the well-labeled source domain (pneumonia) to the partially labeled target domain (COVID-19). Domain discrepancy was minimized via domain adversarial learning and the task difference between domains was eliminated by proposing a novel classifier separation scheme.
CoroNet [53]	[18], [22]	Autoencoders were used for feature extraction.
[57]	[23]	SMOTE was used to handle the class imbalance problem.
[9]	[18]	DarkNet is an end-to-end architecture proposed as a classifier for YOLO real-time object detection. The authors introduced different filters on each layer with an implementation of 17 convolutional layers.
[11]	[18]	Multi-level segmentation thresholding was applied to split grayscale images into areas of intensities to reduce the number of objects in a lung image. An SVM was then used to classify the COVID-19 cases. The MATLAB 2019a DL toolbox was used.
[44]	[18], [65], [69]	The proposed model tuned both the VGG16 and RestNet50 models with a balanced set of CXR images by replacing the last three layers with the trainable part, followed by a 64-unit connected layer with dropout, 10-fold validation, and a classification layer with a sigmoid output.
[84]	[18], [69]	The authors built a framework called discriminative cost-sensitive learning. They stated that this technique should be adopted in clinical settings, such as COVID-19 diagnosis, because it offers two features, i.e., fine-grained classification and cost-sensitive learning.
[80]	[61]	Two types of COVID-19 infections were distinguished using CXR images because the treatment for each type differs significantly. Additionally, this study considered the progression of the disease.
[81]	Locally collected	Proposed a model called Cascade-SEMEnet, which is based on the SENet architecture [47]. Additionally, the study implemented U-Net [98] and introduced adaptive histogram equalization with limited contrast to preprocess and enhance the quality of the images.

### C. Performance Comparisons

It was difficult to compare the studies included in this survey owing to the variations in the size of the testing sets and the lack of standard performance measures, which further complicated the identification of the most efficient DL models for detecting COVID-19 from CXR images. Most authors evaluated the DL models in terms of accuracy, sensitivity, and specificity metrics. However, the difficulty of comparing different approaches increases when non-standard metrics and datasets from multiple sources are used. Thus, it is essential to develop a public COVID-19 dataset that is comprehensive and accessible by the AI research community. In addition, standards for evaluating the performance of prediction models must be established.

Table 5 shows the results of the reviewed articles in terms of the classification metrics such as accuracy, precisions, recall, AUC, and F1-score. The reader can refer to the glossary appendix for the definition of these terms.

**TABLE 5:** Performance Metrics of the Methods Used in the Reviewed Research

Research study	Accuracy (%)	Precision / PPV (%)	Specificity (%)	Sensitivity/ Recall/TPR (%)	AUC	F1-Score
CovidAID [90]		98.60		1	0.99	
[72]	96.90					
[30]	95.30					
Deep-COVID [28]			90.00	97.00		
[73]	89.82					
[74]	93.00	93.15	88.52	97.53	0.95	94.57
COVID-CAPS [31]	95.70		95.80	90.00	0.97	
[26]			87.84	90.00	0.95	
COVID-Net [60]	93.30	98.90		91.00		
DeTraC [36]	95.12		91.87	97.91		
[75]	91.40	35.27	99.56	80.53		
[56]	99.46	99.46	99.73	99.46		99.46
CheXNet [59]	97.80	97.80		97.80		97.80
COVID-DA [62]		98.15		88.33	0.98	92.98
[34]	97.20	97.67	98.51	97.50		97.50
CoroNet [53]	93.50	93.63		93.50		93.51
[76]		99.33		99.33		
[57]	97.70		98.80	97.70	0.98	97.70
[41]	93.90			88.00	0.97	
[8]						
[5]	99.00	99.00		99.80		99.80
[92]		82.00	82.00	78.00	0.89	
[46]	90.00	83.00		1		91.00
[35]	95.38					95.52
[44]	91.24					
[79]	98.26		99.13			
[91]	95.00	97.00		100.0			
[58]	89.78	89.85	99.63	89.79		
[80]	82.70	92.70	62.50			
[81]				80.00		
[82]	97.00					
[42]	89.3	90.00		89.00		90.00

**FIGURE 5.**
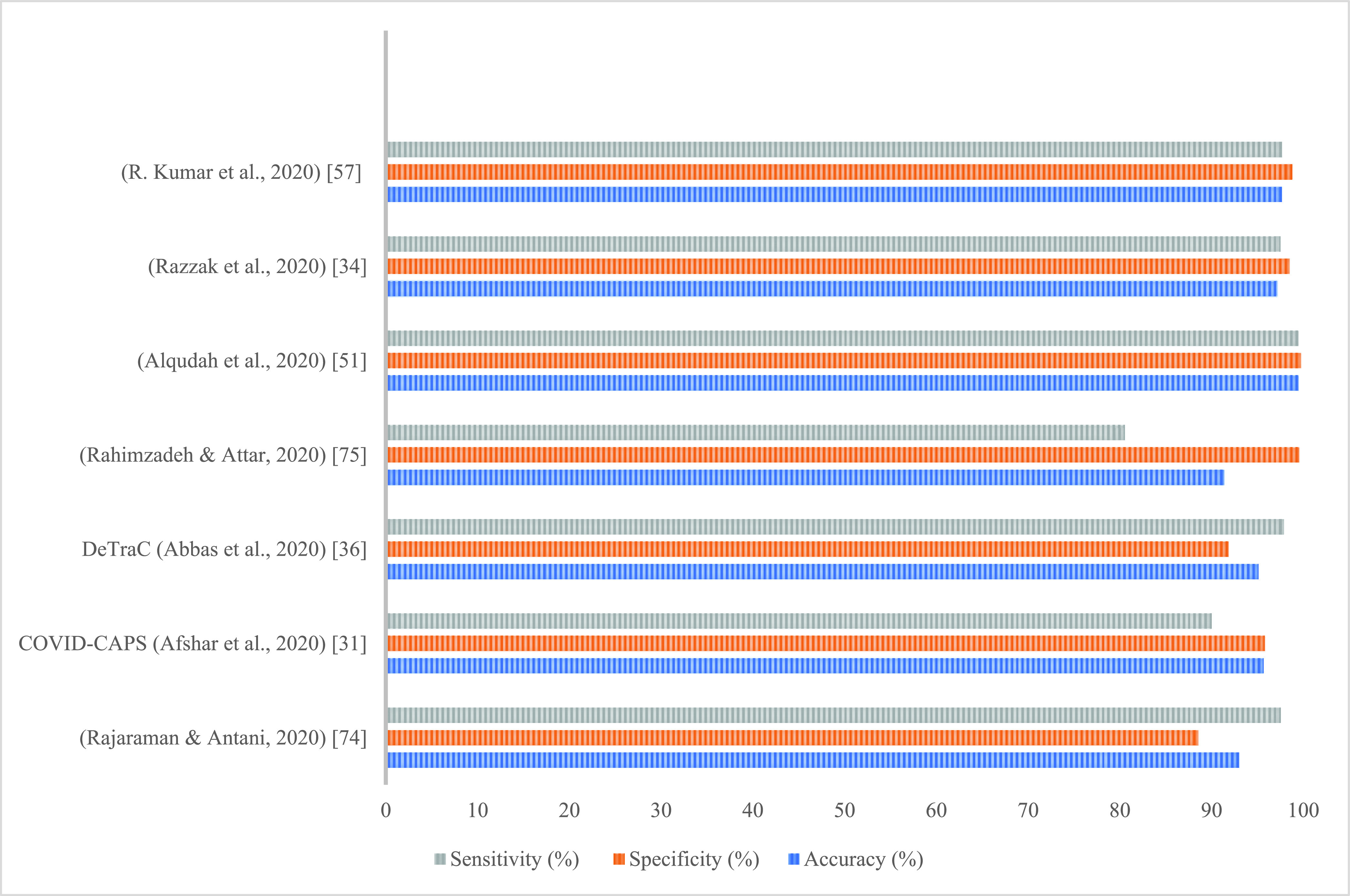
Performance comparison of the results of the reviewed research.

It can be observed from the table that most of the models achieved high accuracies; however, as demonstrated in Table 4, most of these results were obtained over a limited number of COVID-19 samples. Thus, the results are not representative unless the study considered the class imbalance problem in the test set. Fig. 5 illustrates the results of the studies that presented the most common metrics, i.e., accuracy, specificity, and sensitivity. As evident, Alqudah *et al.* [56] achieved the highest performance with respect to all measures. The methods proposed by Razzak *et al.* [34] and Kumar *et al.* [57] also achieved comparable results for all metrics; as demonstrated in Table 5, these two studies dealt with class imbalance by including an equal number of samples for each class and employing the synthetic minority oversampling (SMOTE) technique, respectively.

## IV. Discussions

Despite the encouraging results of the DL architectures, there exist several issues that must be addressed to ensure that the diagnostic process is more accurate, transparent, and trustworthy. In this section, we emphasize the current research challenges associated with the detection of COVID-19 from CXR images.

### A. Class Imbalance Problem

COVID-19 datasets encounter the problem of class imbalance. The uneven distribution of classes raises concerns related to the robustness of the machine learning algorithm. Some studies, such as the study of Kumar *et al.* [57], proposed the use of SMOTE to mitigate this problem. Another approach suggested by Ucar and Korkmaz [78] and Rajaraman and Antani [74] involved the implementation of data augmentation to enlarge the number of images obtained from the limited number of COVID-19 cases. Multiple datasets consist of images containing unrelated visual features and misleading artifacts, which are not usually addressed by the studies covered in this review.

Table 6 lists the number of samples used for the training and testing sets by the studies included in this survey. As shown, the size of the datasets varies widely. COVID-19 cases range from 11 to 1,536 cases, whereas the total sample sizes range from 50 to 224,316 cases.

**TABLE 6:** Sizes of the Datasets Used in the Reviewed Research

Research study	Total samples		Training samples		Testing samples		Method for handling class imbalance
	ALL	COVID-19	ALL	COVID-19	ALL	COVID-19	
CovidAID [90]	3,969	106	3,516	80	424	19	Fixed ratio of classes in each batch
[72]	3,550	864					
[30]	109,895	225					
Deep-COVID [28]	5,071	71	2,031	31	3,040	40	
[73]	5,941	68	4,753		1,188		
[74]	37,220	314	3,883		11,706	314	
COVID-CAPS [31]	94,638	315		284		31	Modification of the loss function
[26]	1,531	100	764	50	767	50	
COVID-Net [60]	13,975	358					Batch re-balancing
DeTraC [36]	196	11	138		58		
[75]	15,085	180	3,783	149	11,302	31	Fixed ratio of classes in each training batch: # samples = 633
[56]	930	310					Equal # samples for each class = 310
CheXNet [59]	2,339	187	2,159	127	180	60	Data augmentation
COVID-DA [62]	11,663	318	10,718	258	945	60	Focal loss
[34]	800	240	800	200	160	40	Equal # samples for each class = 200
CoroNet [53]	18,529	99	16,576	89	1,953	10	Class-weighted entropy loss function
[76]	621	207	366	125	255	82	Equal # samples for each class = 2017
[57]	8,588	62					SMOTE
[41]	327	129					
[8]		127		125			
[5]	9,672	161	7,254		2,418		Data augmentation
[92]	610	324	500	250	110	74	Equal # samples for each class
[46]	50	25	50	20	50	5	Equal # samples for each class
[35]	316	158	0.6		0.2		
[44]	455	135	204	102	251	33	Equal # samples for each class = 102
[79]	5,949	1,536		153		153	Data augmentation
[91]	1,493	284		228		56	
[58]	6,320	464					Class-weighted entropy loss function
[80]	224,316						
[81]	16,130			10		10	
[82]	1,141						New conditional loss, learned with joint balance optimization and cost-sensitive learning
[42]	860	260	580	180	140	40	

As shown, the size of the datasets varies widely. Moreover, the COVID-19 cases are limited and significantly small in number compared to the total number of samples. Thus, several studies considered the class imbalance problem by employing different techniques. Alqudah *et al.* [56] handled this problem by keeping the number of samples in each class equal to 310. Other studies such as [59] and [61] utilized data augmentation. Razzak *et al.* [34], Castiglioni *et al.* [77], El-Din Hemdan *et al.* [46], Hall *et al.* [44], and De Moura *et al.* [76] also considered using a fixed number of samples for each class. Khobahi *et al.* [53] and Duchesne *et al.* [58] used a class-weighted entropy loss function. Kumar *et al.* [57] used the SMOTE technique; Medhi and Hussain [81] and Han *et al.* [83] employed cost-sensitive learning.

### B. Explaining Deep Model Predictions

In most studies covered in this review, DL architectures are used as black-box classifiers, and an explanation of the model decisions is lacking. Explainable AI is an emerging AI subfield that refers to the techniques and methods used to understand the paths taken by machine learning models for decision making. The GSInquire approach [92] was used by Wang and Wong [61] to highlight the areas used by the DL classifier to drive predictions. None of the studies considered defining a region of interest for detecting the symptoms or infections related to COVID-19. However, deep neural network architectures contain numerous optimization parameters; therefore, they heavily rely on large annotated datasets to avoid overfitting.

**FIGURE 6.**
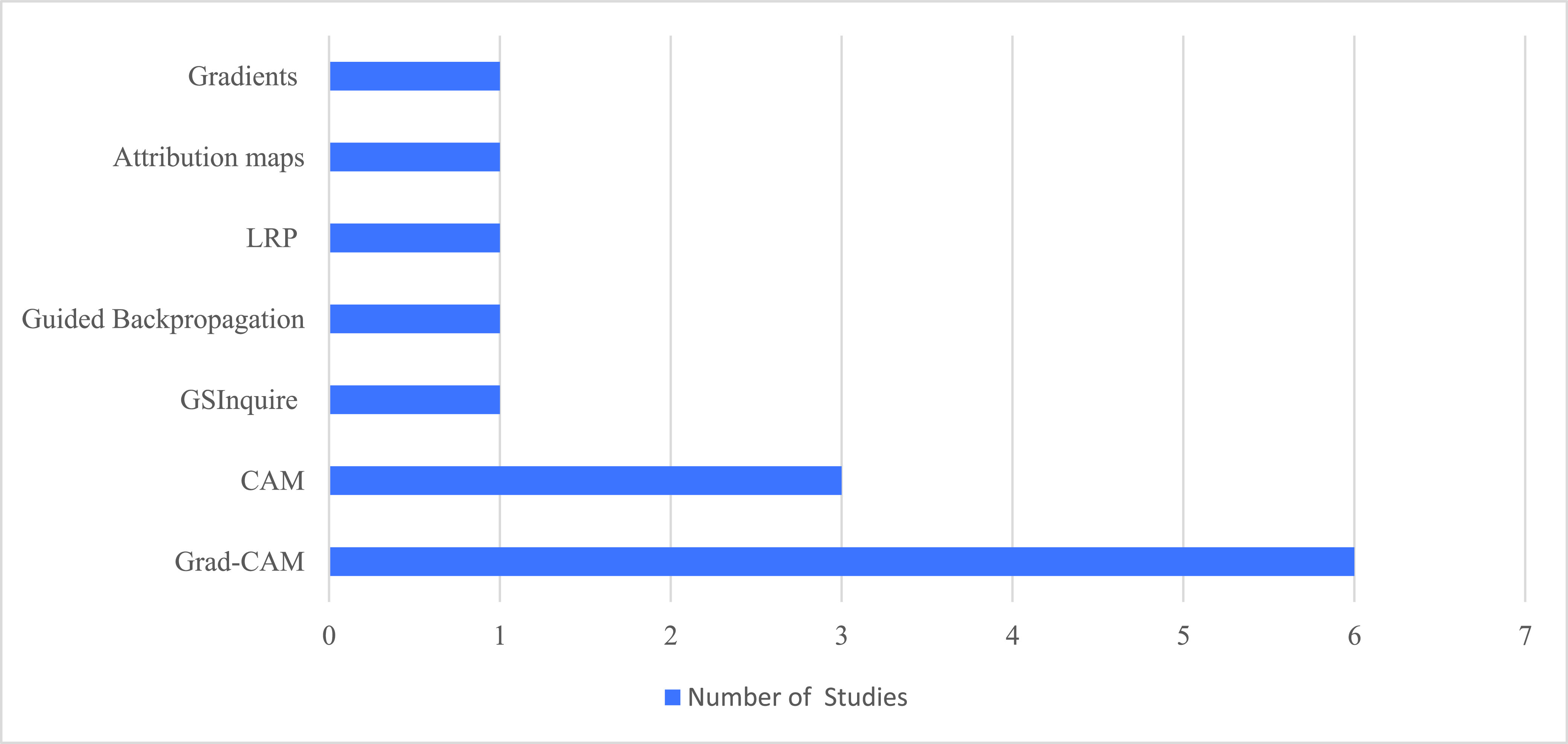
Number of studies utilizing explanatory techniques.

With the increased adoption of the DL models, the demand for explaining how these DL models make decisions has also increased [92]. The lack of transparency and interpretability in DL models hinders their adoption, especially in situations where transparency is crucial, such as in CXR diagnostic scenarios [92]. In the reviewed literature, 11 out of 26 studies (42%) applied DL visualization techniques; Fig. 6 illustrates the number of studies that utilized explanatory techniques. Table 7 summarizes the techniques used and studies that implemented them. As seen in Table 7, the most used method is GRAD-CAM, and next most used is the CAM method. Table 8 shows examples extracted from the reviewed studies showing the explanatory methods used in each work. As illustrated, the GRAD-CAM and CAM methods work in a similar manner, using heat maps, while the other methods each highlight the affected area differently. In the next subsections, we briefly explain each of these techniques.

**TABLE 7:** Methods Used for Explaining Classification Decisions

Visualization technique	Studies utilizing the technique
Grad-CAM	[30], [73], [74], [83], [81]
CAM	[73], [51], [79]
GSInquire	[61]
Guided backpropagation	[73]
LRP	[59]
Attribution maps	[53]
Gradients	[73]

**TABLE 8:** Examples of Explanatory Techniques Used in the Reviewed Studies

Visualization Technique	COVID-19 Case Before Highlighting	COVID-19 Case After Highlighting	Source
Grad-CAM			[30]
CAM			[79]
Gradients			[73]
Guided Backpropagation			[73]
LRP			[59]
Attribution maps			[53]
GSInquire			[61]

#### 1) Grad-CAM

Grad-CAM is the most widely used technique by the reviewed studies, as illustrated in Fig. 6; it was implemented in [30], [73], [74], [26], [82], and [80]. Grad-CAM is a visual explanation technique that distinguishes between classes in the visualization task [93], and it offers several features that might have encouraged the reviewed studies to use it. First, Grad-CAM does not require any changes in the architecture, unlike other techniques such as CAM, which alter the architecture of the CNN [93]. Second, Grad-CAM is highly class-discriminative, i.e., it not only highlights the regions attended to by the classifier during classification but also differentiates between the classes.

#### 2) CAM

CAM is a visualization technique that replaces the fully connected layers with the global average pooling and convolutional layers to create class-specific feature maps because the global average pooling layer enables localization in CNNs [70]. CAM was employed in three studies— [73], [51], and [78]—as shown in Fig. 6.

#### 3) GSInquire

This technique is based on generative synthesis, which is an algorithm that can produce generators that create deep neural networks automatically [92]. This method was adopted by Wang and Wong [61].

#### 4) Guided Backpropagation

In guided backpropagation, the process of finding the discriminative part in an image starts with a high-level feature map. Next, the algorithm reverses the data flow of the network, starting from neuron activation in a given layer back to the image. Consequently, the created image highlights the part of the input image that is responsible for activating the neuron [96]. Guided backpropagation was employed by Ghoshal and Tucker [73].

#### 5) LRP

LRP employs the network weights and the activations created by forward propagation to propagate the output back to the input layer. Thus, pixels that contribute to the output can be visualized [97]. This method was used by Bassi and Attux [59].

#### 6) Attribution Maps

Attribution maps, used by Khobahi *et al.* [53], are heatmaps that show areas in the input image that are responsible for the classification output. To construct an attribution map, a generative model removes parts of the image, where the attribution value of an input area is approximated by the changes in the classification probability [98].

#### 7) Gradients

Gradient is a technique used to visualize a deep convolutional network trained using ImageNet [99]. This method was implemented by Ghoshal and Tucker [73]. A gradient finds the gradient of the class score for the input image and uses backpropagation to represent the gradient as a heatmap.

### C. Managing Classification Uncertainty

Uncertainty in DL represents the level of confidence in the result obtained by the classifier [73]. Obtaining a high softmax output does not imply high certainty, even though the softmax output can be mistakenly confused with model confidence [100] [101]. A DL model that considers uncertainty enables uncertain cases to be treated with caution. Thus, when a model generates a result with high uncertainty, this suggests that human intervention is recommended to examine the result further [78]. Ghoshal and Tucker [73] stated that the estimation of uncertainty in AI solutions can increase their adoption in clinical settings because it provides a more transparent solution that can be trusted by physicians. In the reviewed articles, we found that the study in [73] dealt with model uncertainty. In this research, drop weights and Bayesian CNNs were implemented to compute the uncertainty. The study also implemented transfer learning using a pre-trained ResNet50V2 model finetuned on COVID-19 data. The model generated the output certainty as low or high confidence based on the input CXR image, and the authors illustrated how the confidence level could affect the decision process when using real COVID-19 radiograph examples. Their accuracy ranged between 86.02% and 89.82%. The model showed a relatively high correlation between model uncertainty and prediction accuracy.

According to this review, more studies are required to investigate the uncertainty in the predictions made by a model, which define the level of confidence in the results produced by the model. In classification problems, the data samples can be close to a threshold or decision boundaries, which reduces the confidence accuracy of the classifier regarding the final decision. However, this is not usually discussed in the DL medical imaging literature and was only discussed by Ghoshal and Tucker [73] in the studies included in this review.

### D. COVID-19 Severity Assessment

Other problems that remain to be tackled in the COVID-19 CXR imaging literature include disease progression assessment and prognosis analysis. CXR imaging analysis could also help in identifying high-risk patients and the areas that urgently require attention and support. These issues and problems require more involvement of medical personnel at all stages of DL model development, evaluation, and validation.

Triage is an important stage during the COVID-19 pandemic owing to the growing number of patients who require rapid and accurate intensive care and resources. DL studies that aim to predict, track, and assess the progress and severity of COVID-19 patients help in efficiently triaging patients. Duchesne *et al.* [58] and Islam and Fleischer [79] considered tracking the progress of COVID-19 patients in their studies. Duchesne *et al.* [58] monitored and predicted patient progress using the extracted DL features, which can predict whether a patient’s case would “worsen” or “improve” with an accuracy of 82.7%. Islam and Fleischer [79] used feature-embedded machine learning to distinguish L-type and H-type patients using their CXR images. Moreover, to detect and monitor disease progression and recovery, they categorized multiple images from the same patient.

### E. Training Dataset Quality

Our findings indicate that it is highly likely that some data samples overlap. The same images could be used multiple times in training, particularly when the authors have collected their data from several online resources that include data originally from the same source. One solution to this problem is to run an image similarity assessment process. It is very crucial to detect the amount of duplication in the training and test sets to avoid overusing data samples and overfitting. This problem has not been discussed in any of the works reviewed in this survey.

Annotation is time-consuming and requires radiologists to grade images at pixel level to specify the COVID-19 biomarkers and complications. Currently, the CXR images found in public datasets are only labeled as normal/healthy, pneumonia, and COVID-19. Establishing a dataset annotated with the main characteristics employed by radiologists to derive their decisions would considerably assist in embedding more useful features to a DL model; thus, the transparency of the decisions made by the model can be enhanced and clinically acceptable automatic detection systems can be obtained. The pixel-level annotation of COVID-19 signs would also be beneficial in determining the disease severity for effectively using resources or prioritizing treatment in heavily affected regions.

The lack of uniformity in the CXR images is another problem that must be addressed. Scalable deep neural network classifiers should be built using samples from several diverse resources. However, image diversity introduces the problem of preprocessing efficiency, and more investigations are required in this direction. Moreover, it is essential to ensure that the CXR images are appropriate for automatic analysis. Images should be of sufficient quality and free from misleading features such as descriptive texts or numbers. Therefore, including a quality assessment component in COVID-19 automatic classification systems is highly recommended. This would also enhance the clinical trust toward the computer-aided diagnosis system.

### F. Transfer Learning From General Object Recognition Task

Despite the advantages of employing transfer learning, most of the works used deep models pre-trained on general object recognition tasks such as the ImageNet dataset. Only a few studies, such as Mangal *et al.* [89] and Bassi and Attux [59], used architectures pre-trained on a large dataset of CXR images. However, even these architectures are based on networks originally designed for the ImageNet dataset, which raises several questions about their robustness and effectiveness when used in practical clinical scenarios. The automatic selection and optimization of deep neural network architectures and their hyperparameters is another important research domain that could contribute positively to the COVID-19 classifiers. For instance, a generative synthesis approach was used in [61] by specifying the human design requirements.

## V. Conclusion and Directions For Future Research

This study presents a comprehensive review of the diverse DL methods used to detect COVID-19 from CXR images. The current status of this research is discussed here. Besides, the most common pretrained CNN architectures were explained. The datasets that were utilized by different studies are presented and discussed, and the current challenges associated with the current approaches are highlighted. This survey indicated the significant potential of DL methods in the automatic diagnosis of COVID-19 from the currently available datasets; however, medical personnel and computer scientists should work together closely and utilize their complementary expertise to validate the usefulness of DL techniques.

It was found that CNN-based transfer learning was used in most studies using the same dataset collected by Cohen *et al.* [18]. Despite the encouraging performance achieved, there is still significant room for improvements. First, public, comprehensive, and diverse datasets need to be established. The datasets should be validated by experts and annotated with the corresponding lesions of lung diseases. Incorporating the detection of signs with the classification output would increase both the prediction accuracy and the models’ transparency. Second, as the medical research to determine the main characteristics of COVID-19 is still ongoing, it is essential to utilize more features extracted based on recommendations of medical personnel. Given the small size of available CXR COVID-19 datasets, integrating domain knowledge would help create models that mimic human expert diagnostic patterns and focus on the signs or regions they pay particular attention to. However, appropriate domain knowledge should first be determined. The trade-off between the automatically learned deep features and the extracted domain knowledge features should be managed to achieve the desired performance. Third, it is important to measure the amount of disagreement between radiologists to develop a benchmark for use in the prediction evaluation of the deep learning models. Fourth, considering that clinicians often refer to previous analogous cases to make reliable decisions regarding diagnoses, we believe that semi-supervised learning has great potential yet to be unlocked. Semi-supervised algorithms employ few labeled samples and many unlabeled data as part of the training set. Semi-supervised modelling can not only reduce the cost of data annotation, but also help in discovering hidden patterns and relations in the data. Fifth, as seen, most studies in this survey utilized traditional data augmentation operations to deal with the scarcity of COVID-19 CXR images. The promising results achieved by generative adversarial networks (GAN) are worth further investigation. Finally, the promising results achieved by automatically generating a deep CNN architecture tailored for COVID-19 classification task using GenSynth [60] can also be utilized when researchers make a larger and comprehensive COVID-19 CXR dataset available.

## Appendix

**Overfitting**: This refers to the phenomenon in which a machine learning model learns a function that fits the training samples perfectly with low error but high variance. Such a model would poorly generalize previously unseen samples.

**Regularization**: This technique is used for controlling the overfitting phenomenon by adding an additional penalty term in the cost function to avoid extreme values of the model parameters.

**Data augmentation**: This is a regularization approach that generates an enormous amount of artificial data samples through employing multiple transformations such as flipping, rotating, shifting, resizing, and changing the lighting conditions.

**Transfer learning**: This is a concept based on representation learning with the underlying assumption that some features are common to many different tasks. In this process, a model that has been trained in a certain setting is used to improve generalization in another setting [83].

**Accuracy**: This parameter measures the overall performance of a model. It is calculated as the percentage of the correctly classified data samples by the model.

**Sensitivity, recall, or true positive rate (TPR)**: These parameters measure the number of positive cases correctly predicted by the model.

**Specificity**: This measures the negative cases covered by the model.

**Precision**: This measures the accuracy of the model in predicting positive samples.

**F1-score**: This is the harmonic mean of precision and recall.

**Receiver Operating Characteristic (ROC)**: This curve is a plot that displays the trade-off between precision and recall over a series of cut-off points. The closer the curve to the top left corner, the better the classifier.

**Area Under the Curve (AUC)**: AUC of the ROC is used to evaluate a classifier. For a perfect classifier, the AUC would be equal to one.

**Explainable Artificial Intelligence**: This includes techniques used to visualize and explain DL models, e.g., by highlighting important features in the images used by the model to reach a prediction.

**ImageNet**: This is a benchmark image dataset organized according to the WordNet hierarchy and consists of a collection of 14,197,122 annotated images of numerous everyday objects such as animals, food, devices, and flowers.

**ILSVRC**: The ImageNet Large-Scale Visual Recognition Challenge, which was started in 2010 and ran annually until 2017.
